# Glucosinolates as Markers of the Origin and Harvesting Period for Discrimination of Bee Pollen by UPLC-MS/MS

**DOI:** 10.3390/foods11101446

**Published:** 2022-05-17

**Authors:** Ana M. Ares, Jesús A. Tapia, Amelia V. González-Porto, Mariano Higes, Raquel Martín-Hernández, José Bernal

**Affiliations:** 1 I. U. CINQUIMA, Analytical Chemistry Group (TESEA), Faculty of Sciences, University of Valladolid, 47011 Valladolid, Spain; ana.maria.ares@uva.es (A.M.A.); jesus.tapia@uva.es (J.A.T.); 2 Department of Statistics and Operations Research, Faculty of Sciences, University of Valladolid, 47011 Valladolid, Spain; 3 Instituto Regional de Investigación y Desarrollo Agroalimentario y Forestal de Castilla La Mancha (IRIAF), Centro de Investigación Apícola y Agroambiental (CIAPA), Camino de San Martín, s/n, 19180 Marchamalo, Spain; avgonzalezp@jccm.es (A.V.G.-P.); mhiges@jccm.es (M.H.); rmhernandez@jccm.es (R.M.-H.); 4 Instituto de Recursos Humanos para la Ciencia y la Tecnología (INCRECYT-EFS/EC-FSE), Fundación Parque Científico y Tecnológico de Castilla—La Mancha, 02006 Albacete, Spain

**Keywords:** authentication, bee pollen, bioactive compounds, canonical discriminant analysis, food analysis, food quality, harvesting period, glucosinolates, markers, origin, UPLC-MS/MS

## Abstract

Bee pollen is currently one of the most commonly consumed food supplements, as it is considered to be a good source of bioactive substances and energy. It contains various health-promoting compounds, such as proteins, amino acids, lipids, as well as glucosinolates. In the present study, the glucosinolate content was determined, by means of ultra-performance liquid chromatography coupled to a quadrupole time-of-flight mass detector, in 72 bee pollen samples from four different apiaries in Guadalajara (Spain), harvested in three different periods. In addition, 11 commercial multifloral samples from different Spanish regions were also analyzed. The aim was to verify the suitability of these compounds as biomarkers of their geographical origin, and to test their potential for distinguishing the harvesting period. By means of a canonical discriminant analysis, it was possible to differentiate the apiary of origin of most of the samples, and these could also be clearly differentiated from the commercial ones, simply as a result of the glucosinolate content. In addition, it was also demonstrated for the first time that bee pollen samples were capable of being differentiated according to the time of harvesting and their glucosinolate content.

## 1. Introduction

In the last few years, the consumption of bee products has become increasingly significant, due to an increased public awareness of their nutritional and health benefits. However, their production cannot increase rapidly in the short term, and this can result in substantial economically motivated adulteration. This is compounded by the globalization of supply chains, which has also led to a rise in fraudulent bee products [1]. These illicit products are eroding market prices and consumer trust, whilst causing significant damage to the beekeeping industry. Therefore, authentication of bee products, especially honey and bee pollen, in terms of their genuine botanical and geographical origins, as well as the detection of any adulteration, is essential in order to protect consumer health and to avoid competition that could create a destabilized market [2]. For example, bee pollen from different origins (botanical and geographical) can differ greatly in chemical composition and medicinal functions, and its adulteration will thus seriously interfere with its therapeutic effects and food safety/quality [3]. In recent years, pollen has become one of the most consumed food supplements due to the related health benefits, namely, those of an antioxidant, anti-inflammatory, anticancer, analgesic, antifungal, or antiviral nature [4,5]. This is because pollen contains several health-promoting substances, such as proteins, amino acids, lipids, phenolic compounds, vitamins, or minerals, the specific content of which is greatly dependent on their botanical and geographical origin [4,6]. Therefore, it is not surprising that some of these bioactive compounds (vitamins, proteins, lipids, or phenolic compounds) have been investigated in bee pollen with the aim of evaluating their potential as quality indicators, or as markers of its origin [7,8,9,10,11]. However, until a few years ago little attention had been paid to a family of compounds in bee pollen, namely, glucosinolates, which were extensively studied in plant matrices, along with their derivatives, due to their antioxidant, antifungal, or anticancer activities [12,13]. Glucosinolates (GSLs) are natural compounds of the secondary metabolism of plants, mainly present in dicotyledonous plants and especially abundant in the Brassicaceae (cruciferous) family [14]. These compounds have been previously proposed as potential botanical biomarkers, not only of Brassicaceae plants [15] but also of honey [16] and, more recently, in bee pollen by our research group [17]. This latter study has demonstrated the potential of GSLs as biomarkers of bee pollen origin, as it was possible to differentiate the apiary of origin on account of the glucosinolate content of their corresponding bee pollen samples. However, the number of these in some of the apiaries was not large, and they were selected according to their different colors, as pollen balls from each sample were grouped by color. Thus, the aim of the present study is to confirm our previous findings by determining the GSL content, by means of ultra-performance liquid chromatography coupled to a quadrupole time-of-flight mass spectrometer (UPLC-QTOF/MS; [18]), in a larger number of bee pollen samples from the same four apiaries (Fuentelahiguera, Monte, Pistacho, Tío Natalio) located in the same geographical area (Marchamalo, Guadalajara, Spain). Regarding another aspect in respect of our previous study, there was no prior separation by color, and harvesting took place in three consecutive foraging periods (April–May; June; July–August). Additionally, GSL content was also determined in commercial bee pollen samples so as to compare them with the bee pollen samples from Marchamalo. This comparison was conducted in all cases by using chemometrics, or, more specifically, canonical discriminant analysis (CDA). To the best of our knowledge, this study represents an analysis of the largest number of bee pollen samples for determining GSL content ever carried out, and the first time that a potential relationship of GSL content with the bee pollen harvesting period has been studied.

## 2. Materials and Methods

### 2.1. Chemical and Materials

GSL standards (Det. Purity > 95%; see Table 1) were obtained from Phytoplan Diehm & Neuberger GmbH (Heidelberg, Germany). All reagents were of LC grade [17,18,19], while the devices/instruments employed in the present study are summarized in Table S1 (see Supplementary Materials).

### 2.2. Standards

Individual standard stock (≈100 mg/L) and working solutions were prepared with ultrapure water. Matrix-matched standard calibration curves (limit of quantification; LOQ; see Table 1—1500 μg/kg) were employed to quantify GSLs due to the matrix effect that affected their MS/MS signals [17,18]. Limits of detection (LODs) and LOQs were experimentally estimated as three and ten times the signal to noise ratio, respectively [18].

### 2.3. Sample Procurement and Treatment

#### 2.3.1. Samples

**Figure 1:**
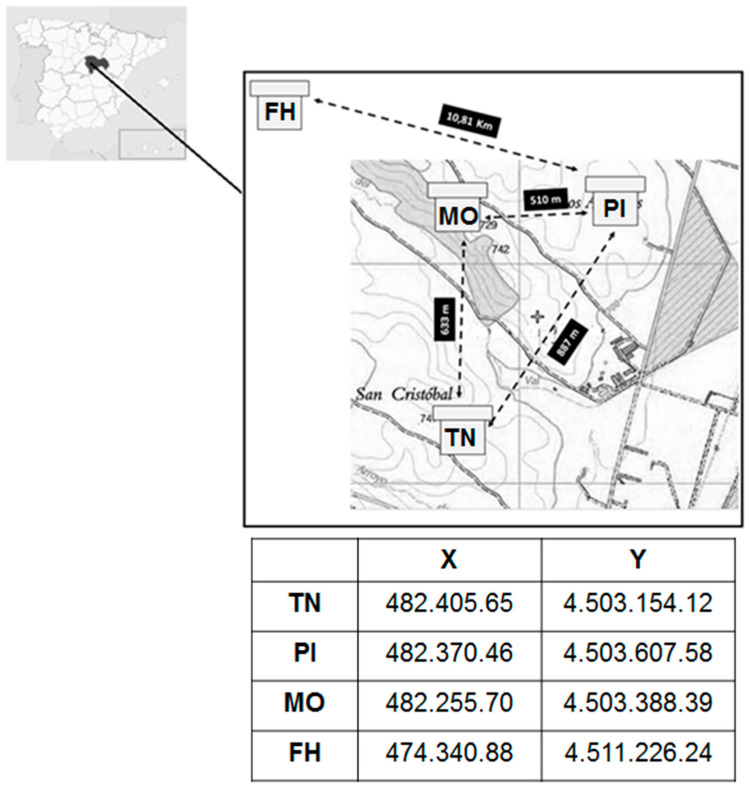
Location and Global Positioning System (GPS) data of the apiaries (Fuentelahiguera, FH; Pistacho, PI; Monte, MO; Tio Natalio, TN).

Bee pollen samples were obtained from four apiaries with homogeneous colonies of *Apis mellifera iberiensis* (*n* = 72) and from local markets in Valladolid (Spain; *n* = 11). All the commercial samples were labelled as multifloral, and the specific geographical origin was not provided, as it was only stated that they were produced in Spain. Three of the apiaries, Pistacho (PI), Tío Natalio (TN), and Monte (MO), were located on the Centro de Investigación Apícola y Agroambiental (CIAPA) farm in Marchamalo, and the fourth, Fuentelahiguera (FH), was in the municipality of Fuentelahiguera de Albatages; all of them were in the province of Guadalajara (Spain; see Figure 1). The PI apiary consisted of 20 colonies arranged in a plot in which the plant species were for spring and autumn cultivation. The higher number of colonies (20) was determined by the existence of several influential factors in diversity, such as the varied type of crops present, different for each period. All the crops were selected because they are highly attractive to bees and other pollinators, they possessed a potentially high nutritional value for these insects, and because of their significant economic importance in our country, two of the main sources being rapeseed (*Brassica napus*) and vetch (*Vicia sativa*). The hives were distributed in two rows in front of the crop and in a central lane, in order to facilitate the access of the bees to the crops. The blooms were staggered according to the plant species, beginning in April. In each of the other three apiaries (TN, FH and MO), five colonies were selected for the study, and at least three samples were available per collection period, so that the results and interpretation were adequate. Those apiaries were located in an area primarily occupied by wild vegetation, although their proximity to the crops, especially in TN, did not exceed 3 km. The number of colonies in these apiaries was only five, since it is classically considered sufficient, and because the only determining factor of variability in diversity was related to the succession of flowering of the plants present in the surroundings of these apiaries. Bee pollen samples were collected using pollen traps placed at the entrance of the hive. Every two weeks, the pollen trap grid was closed for a period of 24 h in the different hives. The pollen stored in the collection drawer during this period was collected, and immediately taken to the laboratory, where it was frozen until palynological analysis. In this study, the bee pollen samples were collected during three consecutive foraging periods; these are defined as initial (between April and May), intermediate (June), and final (between July and August). The colony of origin and harvesting period for each bee pollen sample is summarized in Table S1 (see Supplementary Materials). Finally, it should be explained that the fact of not using all of the samples collected in this study is related to not having the necessary amount to address all of the analyses in each harvesting period, due to a lower amount of pollen collected by the bees.

#### 2.3.2. Palynological Analysis

This was performed as previously described [17,20], the difference being that the pollen balls were not separated by color. In the present study, a representative fraction of each bee pollen sample (≈20 g) was subjected to palynological analysis. Briefly, pollen was extracted by diluting 0.5 g in 10 mL of water and then centrifuged at 2500 r.p.m. for 15 min. Next, the sediment was poured onto the glycerin jelly slide after removal of the supernatant and examined under a microscope to identify the pollen (×250 magnification, Leitz Diaplan microscope, Leitz Messtechnik GmbH, Wetzlar, Germany). The species of plant was identified by means of a photographic atlas [19], together with the reference collection of pollen slides from CIAPA [20]. The results of the contents of corbicula pollens, mostly collected in the samples and corresponding to each period and colony, are summarized in Table 2.

Whenever the majority taxon within the composition of the collected sample was well defined (greater than 50%), the name of that taxon was applied in the corresponding column, while in those samples where this requirement was not met, the denomination MF (multifloral) was applied. MF terminology, which is based on specialized literature [21,22], relates to pollen compositions in which there is no single main or majority taxon, but a combination of these in the sample, not totaling more than 45%.

#### 2.3.3. Sample Treatment

Bee pollen samples were treated with the same procedure that was previously developed and optimized by Ares et al. [17,18,23]. The steps of this procedure are detailed in Table S2 (see Supplementary Materials). It must be specified that following this sample treatment, GSLs maintained the sulfate group in their structure, and for this reason they are specifically known as intact-GSLs [24].

### 2.4. UPLC-MS/MS System

An Acquity™ UPLC system (ACQUITY, Waters, Milford, MA, USA) and a Q-TOF/MS spectrometer (maXis impact, Bruker Daltonik, Bremen, Germany) were coupled through an electrospray (ESI, negative mode) interface, which was operated in the negative ionization mode. UPLC-MS/MS conditions were taken from our previous study [19]. A Luna^®^ Omega C_18_ column (50 × 2.1 mm, 1.6 μm) and guard column (Phenomenex, Torrance, CA, USA) were employed for all UPLC analyses. Mobile phase composition was described in Table S2 (see Supplementary Materials); meanwhile, injection volume and column temperature were set at 5 μL and 30 °C, respectively. In addition, the values of the most relevant MS/MS parameters were listed in Table S2 (see Supplementary Materials). The ions monitored for each compound are summarized in Table 1. The chromatogram of a bee pollen sample (PI-11) obtained using the selected UPLC-MS/MS conditions is shown in Figure S1 (see Supplementary Materials).

### 2.5. Canonical Discriminant Analysis

The calculations for CDA required in this paper were performed using SAS PROC CANDISC (version 9.4; SAS Institute Inc., Cary, NC, USA). Database observations can be classified in two or more groups, and in each group the same quantitative information is measured. Next, statistical analysis detects that the mean vectors are different in the groups. CDA obtains linear combinations of the quantitative variables that emphasize the differences among the groups [17,25]. CDA calculates so many canonical variables as quantitative variables have been measured in the sample, and all of the canonical variables explain all of the original inter-group variability in data. To determine how many canonical variables must be used in the CDA, one should consider the possible proportion of accumulated variability explained by the canonical variables, at least 90%. The database used in the present study comprised the response of each sample to the qualitative variable (apiary of origin or harvesting period), and the three analyses of each individual sample for each GSL (quantitative variables).

## 3. Results and Discussion

### 3.1. Glucosinolate Content

GSL content was determined in the 72 samples of bee pollen from the four apiaries located in Marchamalo (PI, *n* = 45; MO, *n* = 12; TN, *n* = 8; FH, *n* = 7) and 11 samples, purchased in three different local supermarkets (Valladolid, Spain); these corresponded to the same season, and were of multifloral origin and from various regions of Spain. On the labels of the commercial samples the only information provided concerned the botanical (multifloral) and geographical (regional) origins. All the samples were analyzed in triplicate, and in some cases it was necessary to dilute the extracts with ultrapure water; the ratio depended on the sample and GSLs (1:200, 1:300, 1:400; *v*/*v*), due to the high concentrations being outside the linear range. The results are given in Table 3, Table 4 and Tables S3–S11 (see Supplementary Materials), in which the frequency and concentration intervals are shown.

As can be seen in Table 3, GIB was the only GSL that was not detected in any sample from the experimental apiaries; meanwhile, the other GSLs were detected in at least 8 of the 72 samples. GBN, GNA, NEO, and NAS were found in over 50% of these samples, and the highest concentrations were observed for PRO, ALY, NAS, GBN, and NEO (>5500 µg/kg). Regarding the commercial samples (see Table 4), the highest frequencies encountered corresponded to 4-OH and GNA, with 100% and 91%, respectively. In addition, GSLs were detected at higher concentrations along with GTL, but, with the exception of 4-OH, the values were always lower than 1600 µg/kg. Certain significant differences in GSL content were observed between both groups of samples: (i) PRO and GBN were not detected in any of the commercial samples, while they were present in more than 30% of the apiary samples and at some of the highest concentrations; (ii) 4-OH was found in all of the commercial samples at the highest concentrations (>4000 µg/kg), yet was determined in fewer than 35% of the apiary samples at lower concentrations (<3400 µg/kg); (iii) GTL was barely detected in these samples (<12%), whilst it was one of the most commonly observed GSLs in the commercial ones (>60%). These findings could be tentatively related either to the different origin of commercial samples, which was not specified on the labels, and which corroborates our previous results [17], or to the different storage and processing conditions that could affect the stability of GSLs. Nevertheless, the detection of a GSL (4-OH) in all of the commercial samples implies that they contained pollen from Brassicaceae plants. Regarding the results obtained from the four apiaries, the following observations could be made: (i) In the MO apiary (see Supplementary Materials, Tables S4 and S5), GIB and GTL were not detected in any of the samples; meanwhile, GBN (in addition to an 83% frequency rate), NAS (50%), PRO (42%), NEO (75%), and ALY (33%) were detected at higher concentrations; (ii) In the PI apiary (see Supplementary Materials, Tables S6 and S7), GIB was not detected in any of the samples, and GTL was detected in 13% of these, albeit at low concentrations. GSLs found at higher concentrations were NAS (44%), GBN (76%), ALY (29%), PRO (36%), and NEO (60%), results which are quite similar to those obtained for the MO apiary; (iii) In the TN apiary (see Supplementary Materials, Tables S8 and S9), GIB, PRO, GRA, and 4-OH were not detected. The number of samples was much lower than that of the previous apiaries, and the GLS concentrations and frequencies were the lowest of the four apiaries (<600 µg/kg; <40%).

This could be related to the multifloral origin of most of the samples; (iv) In the FH apiary (see Supplementary Materials, Tables S10 and S11), GIB, GTL, and 4-ME were not detected in any sample, whilst NEO (71%), 4-OH (29%), NAS (86%), PRO (43%), GBN (43%), and ALY (43%) were detected in higher concentrations (appearance frequency). These findings are in good agreement with those of previous studies [17,18,26], in that, for example, PRO was the predominant GLS in most cases. This is associated with this compound being usually found in rapeseed, a crop which is common in the areas close to the apiaries. The absence of GIB in the samples analyzed is also reported in the above-mentioned publications. Moreover, EPI was detected in samples from the four apiaries, but always at a very low rate of frequency and at low concentrations. A similar comment could be made regarding GTL, which was found in only two of the four apiaries, and also at very low concentrations. Neither of these GSLs usually appear in matrices, similar to rapeseed or radish. Finally, it should be pointed out that a large number of the samples were of multifloral origin, which is why it is also difficult to reach more specific conclusions in relation to GSL content in the corresponding samples and their botanical origin. In addition, differences between the samples from the same apiary could be attributed to the predominant flowering plants in the particular harvesting periods (see Section 2.3).

### 3.2. Canonical Discrimant Analysis

As previously mentioned, all of the bee pollen samples were injected into the UPLC-MS/MS in triplicate; in this way, it was possible to find the differences between the origin (commercial and experimental apiaries) or the harvesting period by using the response to the quantitative variables (GSLs) corresponding to the three measurements made and the concentration confidence interval. If the mean (average value) of the three measurements of each sample were used, instead of the three different values, the variability of the original data would be greatly affected, which is not advisable when performing a CDA.

#### 3.2.1. Origin

##### All Samples

**Figure 2:**
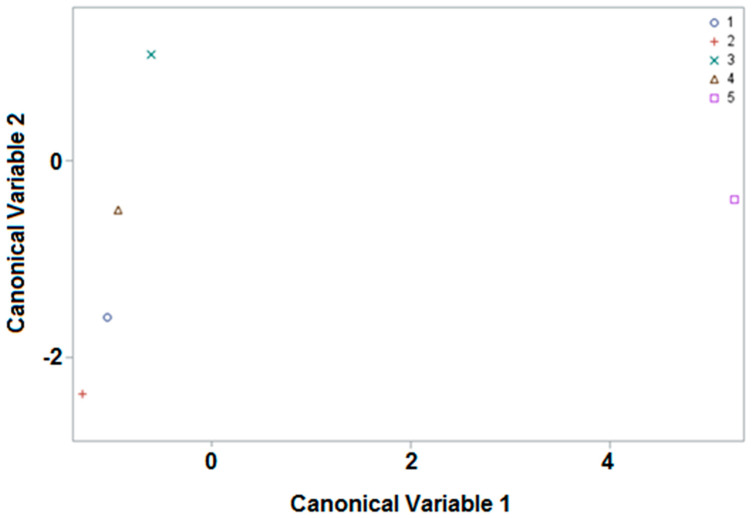
Representation of the apiaries (FH, 1; MO, 2; PI, 3; TN, 4) and the commercial samples (5) as a function of the first two canonical variables.

Initially, there were 83 samples (72 from Marchamalo and 11 commercial samples), 42 variables (14 GSLs present in some of the samples in triplicate), and 5 classes (4 apiaries and commercial samples). As can be seen, there were many variables, and with our statistical approach we can reduce dimensions without losing information to permit a feasible graphic representation in two or three dimensions. Firstly, by considering a classification variable and several quantitative factors, the weights of the canonical variables are obtained; these are linear combinations of the quantitative variables. These values are found in Table S12 (see Supplementary Materials). It should be noted that, for example, SIN_1, SIN_2 and SIN_3 refer to the values obtained in each of the three analyses that were carried out on each sample of the bee pollen. The first four canonical variables reflect the entire variability of the original data, although the first three variables are sufficient to explain more than 90% of the variability. With these values, the average of scores (projections in the canonical functions) of these canonical variables for the five classes were obtained (see Supplementary Materials, Table S13), and the mean of canonical variables 1 and 2 were represented; significant differences were visible between the apiaries and the commercial samples. The position of the points represented depends on the weight (positive or negative value) of the canonical variables of the different GSLs. When canonical variable 2 represented on the ordinate axis is high, it means that the positive values of the variables will have a greater weight in the table than the negative ones, and when the variable is lower, the negative values of the variables will have a greater weight. Meanwhile, for canonical variable 1, represented on the abscissa axis, the positive values will have a greater weight than the negative ones when the value appears on the right, and when this is more to the left, the negative values will have a greater weight. Taking this into account, in Figure 2 it can be seen how the commercial samples (CO) can be clearly differentiated from the rest of the apiaries, using only canonical variable 1; this is because it is the only one that acquires positive values (right side of the abscissa axis).

Moreover, the PI apiary can be differentiated from the other three apiaries by situating the mean of its values in the upper part of the ordinate axis (positive values), whilst the mean values of the other three apiaries are represented in the lower part (negative values).

In addition, if the response of each sample is studied individually (see Supplementary Materials, Figure S2), differences can be observed between the distribution of the canonical variables of the represented samples in accordance with the origin. In addition, a CDA was performed using the linear discriminant function, with the results shown in Table S14 (see Supplementary Materials); it can be observed that many of the samples could be correctly classified: 57% (4 of 7), FH apiary; 91% (11 of 12), MO apiary; 71% (32 of 45), PI apiary; 100% (8 of 8), TN apiary; 100% (11 of 11), CO samples. We can appreciate 100% accuracy in discriminating the commercial samples from the four apiaries, which corroborates the potential of GLSs as biomarkers of the sample origin. To the best of our knowledge, this is the first time that samples from different geographical origins have been differentiated by means of GLS content. The results could be considered satisfactory, because there is over 50% mapping accuracy, which means that the model is sufficiently acceptable. The reason for some samples not being classified correctly may be fundamentally due to most of these being multifloral; consequently, there is a great difference in terms of plant origin.

##### Apiary Samples

**Figure 3:**
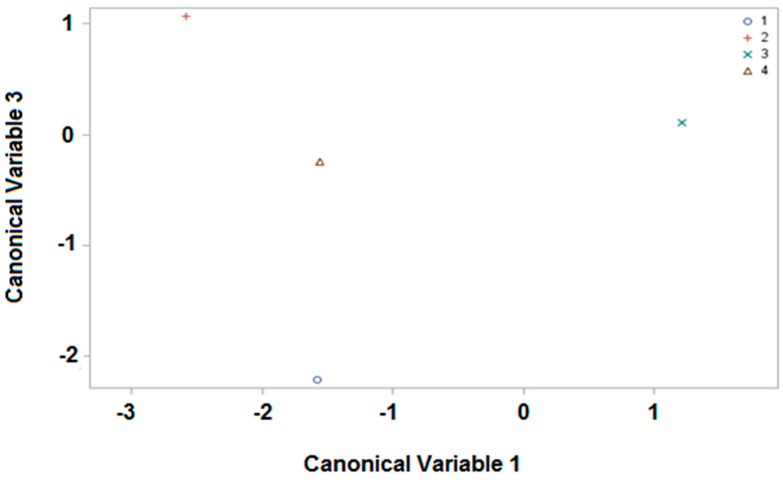
Representation of the apiaries (FH, 1; MO, 2; PI, 3; TN, 4) as a function of canonical variable 1 and 3.

Once the samples corresponding to the commercial group (CO) had been clearly differentiated from those of the apiaries, we performed a different statistical analysis with the 72 samples from the four apiaries. In this case, the sample size was 72, with, once again, 42 variables, and the number of classes (apiaries) was now 4. The weights of the canonical variables are summarized in Table S15 (see Supplementary Materials). The first three canonical variables were now sufficient to account for the 100% variability in the original data. With these values, the average of the scores of the four apiaries studied were obtained (see Supplementary Materials, Table S16) and graphically represented. In this case, the representation of canonical variables 1 and 3 is shown (see Figure 3), instead of 1 and 2 as before; here, an even more distinct separation of the mean values for each apiary was obtained.

However, the basis is the same, and with only two canonical variables a very high degree of variability in the original data can be explained. There is an appreciable differentiation of the four apiaries depending on the positive or negative position of the mean values of the samples in accordance with the canonical variable, 1 or 3. In addition, if the response of each sample to the first three canonical variables is studied individually (see Supplementary Materials, Figure S3), differences can be observed between the distribution of the canonical variables of the represented samples in line with the apiary of origin. A CDA was also performed using the linear discriminant function, with the results shown in Table S17 (see Supplementary Materials). These were very good, as over 70% mapping accuracy was obtained (71% (5 of 7), FH apiary; 83% (10 of 12), MO apiary; 86% (39 of 45), PI apiary; 100% (8 of 8), TN apiary). These results are even better than those obtained with the inclusion of the commercial samples, and this might be explained by the smaller sample size and sample variability. Nevertheless, these results have confirmed the potential of GSLs as markers of the geographical origin, as a large number of the samples could be correctly attributed to apiaries in the same area. Unlike our previous study, these were not previously separated on the basis of color and predominant botanical origin.

#### 3.2.2. Harvesting Period

**Figure 4:**
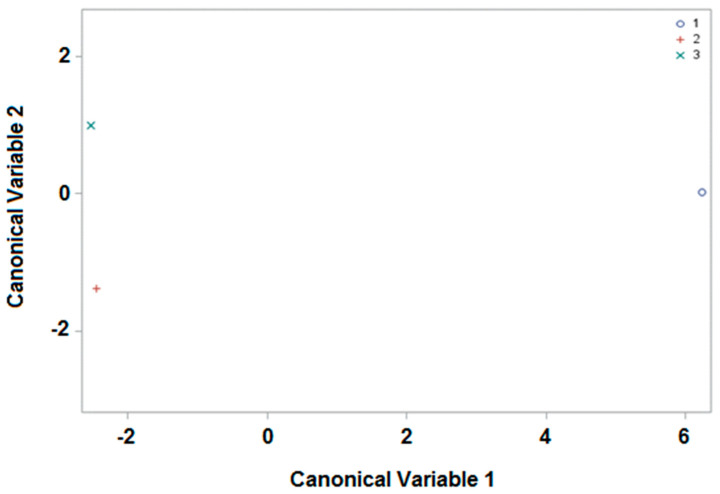
Representation of the harvesting periods (April–May, 1; June, 2; July–August, 3) as a function of the first two canonical variables.

Finally, we attempted to ascertain whether the samples from the apiaries could be differentiated in accordance with GLS content and their corresponding harvesting period (see Table 1 and Section 2.3.1). Sample size and the number of variables remained the same as in Section 3.2.1., but due to classification by harvesting period rather than by apiaries, there were three classes. The weights of the variables are summarized in Table S18 (see Supplementary Materials), and on this occasion, with only the first two canonical variables, the 100% variability in the original data can be explained. Consequently, with these values the average scores of the first two canonical variables were obtained (see Supplementary Materials, Table S19), and these are represented in Figure 4 and Figure S4 (see Supplementary Materials). As can be observed, the separation of the bee pollen samples was even better when considering the harvesting period than with reference to their origin. For example, the average values corresponding to the period “April–May” acquired positive values for canonical variable 1, which differed from those corresponding to the other two periods with negative values. These two periods can also be discriminated, as the mean values of the samples corresponding to “June” are represented above on the ordinate axis (positive values for canonical variable 2), while those of “July–August” represent the negative values of the same canonical variable.

Finally, if canonical discriminant analysis is studied using the linear discriminant function (see Supplementary Materials, Table S20), it may be concluded that more than 60% of the samples could be correctly assigned (100% (20 of 20), April–May; 65% (19 of 29), June; 95% (20 of 21), July–August). This is considered a success from a statistical point of view, and it also represents a relevant finding, as it is the first time that bee pollen samples have been differentiated because of their GLS content and the harvesting period, which could increase the potential of these compounds as pollen biomarkers.

## 4. Conclusions

Intact GSLs were determined by UPLC-MS/MS in 83 bee pollen samples from apiaries located in Marchamalo (Guadalajara, Spain; *n* = 72) and from local supermarkets (Valladolid, Spain; *n* = 11). This represents the largest study carried out to date with these compounds in this matrix. GSLs were found in most of the samples analyzed, although these differed in terms of number and concentration. Only one of the GSLs under study (GIB) was not detected in any of the 83 samples. It was possible to differentiate the commercial samples from the others (MO, PI, TN, and FH), by means of a CDA based on their GSL content and by using the first four canonical variables to reflect 100% variability in the original data. In addition, the samples corresponding to the four apiaries could be differentiated from each other by means of only the first three canonical variables. It has been demonstrated for the first time that bee pollen samples may be classified according to their harvesting period by studying the GSL content. This was achieved by use of only the first two canonical variables, which is a simpler option than the one followed for determining origin. Briefly, it was possible to classify a large number of bee pollen samples according to their origin and harvesting period by means of CDA using the GSL content as the statistical (quantitative) variable. These results not only reinforce the role of GSLs as biomarkers of the bee pollen origin, but also offer a new perspective regarding their significance. It has been demonstrated that these may be key to determining the harvesting periods related to the corresponding predominant flowering plants, which may help to increase the traceability of the bee pollen samples. Finally, the potential applicability of this work is not limited to the bee pollen samples from Spain, but to every country producing bee pollen with Brassicaceae plants on its territory.

## Figures and Tables

**Table 1:** UPLC-MS/MS data and limits of detection and quantification for the studied GSLs. Adapted with permission from Ref. [18]. 2019, Springer Nature.

Compound Name (Abbreviation)	Molecular Weight	Ions (*m/z*)	LOD (µg/kg)	LOQ (µg/kg)
Glucoiberin (GIB)	423	422.0044 ^A,QC^, 96.9540 ^B,C^	18	60
Progoitrin (PRO)	389	388.0203 ^A,QC^, 96.9543^B,C^	18	60
Sinigrin (SIN)	359	358.0120 ^A,QC^, 96.9544 ^B,C^	16	52
Epiprogoitrin (EPI)	389	388.0283 ^A,QC^, 96.9539 ^B,C^	12	38
Glucoraphanin (GRA)	437	436.0291 ^A,QC^, 96.9537 ^B,C^	14	44
Gluconapin (GNA)	373	372.0269 ^A,QC^, 96.9542 ^B,C^	7	23
Glucoalyssin (ALY)	451	450.0500 ^A,QC^, 96.9541 ^B,C^	16	55
4-hydroxyglucobrassicin (4-OH)	464	463.0337 ^A,QC^, 96.9540 ^B,C^	25	80
Glucobrassicanapin (GBN)	387	386.0379 ^A,QC^, 96.9544 ^B,C^	8	26
Glucotropaeolin (GTL)	409	408.0224 ^A,QC^, 96.9542 ^B,C^	7	21
Glucoerucin (GER)	421	420.0400 ^A,QC^, 96.9536 ^B,C^	10	32
Glucobrassicin (GBC)	448	447.0348 ^A,QC^, 96.9542 ^B,C^	9	34
Gluconasturtiin (NAS)	423	422.0401 ^A,QC^, 96.9543 ^B,C^	6	19
4-Metoxyglucobrassicin (4-ME)	478	477.0403 ^A,QC^, 96.9538 ^B,C^	28	88
Neoglucobrassicin (NEO)	478	477.0440 ^A,QC^, 96.9540 ^B,C^	8	25

^A^ Precursor ions; ^B^ Product ions; ^QC^ Quantification and Confirmation ions; ^C^ Confirmation ions.

**Table 2:** Major taxon and harvesting period data of the bee pollen samples from four different apiaries (Monte, MO; Pistacho, PI; Tío Natalio, TN; Fuentelahiguera, FH).

Sample	Major Taxon	Harvesting Period
MO-1	Brassica t. + MF	April–May
MO-2	Brassica t.	April–May
MO-3	Quercus	April–May
MO-4	Brassica t.+ MF	April–May
MO-5	Papaver + Rubus	June
MO-6	MF	June
MO-7	Papaver + MF	June
MO-8	MF	June
MO-9	Papaver + Retama t.	June
MO-10	MF	July–August
MO-11	Rosa t.	July–August
MO-12	MF	July–August
PI-1	Brassica t.	April–May
PI-2	Brassica t.	April–May
PI-3	Brassica t.	April–May
PI-4	Brassica t.	April–May
PI-5	Brassica t.	April–May
PI-6	Brassica t.	April–May
PI-7	Brassica t.	April–May
PI-8	Brassica t.	April–May
PI-9	Brassica t.	April–May
PI-10	Quercus ilex t.	April–May
PI-11	Brassica t.	April–May
PI-12	Brassica t.	April–May
PI-13	Brassica t.	April–May
PI-14	MF	June
PI-15	MF	June
PI-16	Papaver + Rosa t.	June
PI-17	Teucrium + Rosa t.	June
PI-18	MF	June
PI-19	MF	June
PI-20	Rubus	June
PI-21	Papaver + Rosaceae	June
PI-22	Reseda + Retama t.	June
PI-23	Reseda	June
PI-24	Papaver + Retama t.	June
PI-25	Cytisus t.	June
PI-26	MF	June
PI-27	MF	June
PI-28	MF	June
PI-29	Papaver + Rosa t.	June
PI-30	MF	June
PI-31	MF	June
PI-32	MF	July–August
PI-33	MF	July–August
PI-34	MF	July–August
PI-35	Rosa t.	July–August
PI-36	MF	July–August
PI-37	MF	July–August
PI-38	Rosa t.	July–August
PI-39	MF	July–August
PI-40	Retama t.	July–August
PI-41	MF	July–August
PI-42	MF	July–August
PI-43	MF	July–August
PI-44	Rubus	July–August
PI-45	MF	July–August
TN-1	MF	June
TN-2	MF	June
TN-3	MF	June
TN-4	MF	June
TN-5	MF	June
TN-6	MF	July–August
TN-7	MF	July–August
TN-8	Rosa t	July–August
FH-1	Brassica t.	April–May
FH-2	MF	April–May
FH-3	Brassica t.	April–May
FH-4	Vicia t.	June
FH-5	Rosaceae	June
FH-6	Rosa t.	July–August
FH-7	Rosa t.	July–August

^MF^ Multifloral.

**Table 3:** Overall frequency and concentration range of each GSL in the bee pollen samples from the four apiaries.

GSL	Frequency ^A^ (%)	Concentration Range * (µg/kg; Dry Weight)
GIB	0	<LOD
PRO	33	130–6690
SIN	33	84–2721
EPI	18	41–55
GRA	26	47–1172
GNA	56	27–1354
ALY	29	143–7916
4-OH	32	105–3370
GBN	69	31–8469
GTL	11	22–76
GER	26	34–152
GBC	26	39–1467
NAS	47	19–9936
4-ME	26	109–1664
NEO	61	29–5562

^A^ (Number of samples in which a GSL residue was detected/total number of samples (*n* = 72)) × 100; <LOD, below the limit of detection; * Concentrations over the limit of quantification (LOQ).

**Table 4:** Overall frequency and concentration range of each GSL in the commercial bee pollen samples.

GSL	Frequency ^A^ (%)	Concentration Range * (µg/kg; Dry Weight)
GIB	0	<LOD
PRO	0	<LOD
SIN	9	92
EPI	36	57–557
GRA	36	69–249
GNA	91	49–1216
ALY	9	<LOQ
4-OH	100	639–4193
GBN	27	<LOQ
GTL	64	23–1593
GER	0	<LOD
GBC	27	73–419
NAS	45	23–623
4-ME	27	<LOQ
NEO	18	<LOQ

^A^ (Number of samples in which a GSL residue was detected/total number of samples (*n* = 11)) × 100. <LOD, below the limit of detection; <LOQ, below the limit of quantification; * Concentrations over LOQ.

## Data Availability

The datasets generated during the current study are contained within this article and the Supplementary Materials, or they are available from the corresponding author on reasonable request.

## Supplementary Materials

The following supporting information can be downloaded at: https://www.mdpi.com/article/10.3390/foods11101446/s1, Figure S1: UPLC-MS/MS extracted ion chromatogram obtained in positive mode using the quantification ions (see Table 1) from PI-11 bee pollen sample in which eleven GSLs were detected: PRO (1); SIN (2); GRA (3); GNA (4); ALY (5); GBN (6); GER (17); GBC (8); NAS (9); 4-Me (10); NEO (11). The UPLC-MS/MS conditions are summarized in Section 2.4 and Table 1; Figure S2: Representation of individual bee pollen samples (see Table 2) from the apiaries (FH, 1; MO, 2; PI, 3; TN, 4) and local markets (5) as a function of the first two canonical variables; Figure S3: Representation of individual bee pollen samples (see Table 2) from the apiaries (FH, 1; MO, 2; PI, 3; TN, 4) as a function of canonical variables 1 and 3; Figure S4: Representation of individual bee pollen samples (see Table 2) from the different harvesting periods (April–May, 1; June, 2; July–August, 3) as a function of the first two canonical variables; Table S1: Colony of origin and harvesting period for each bee pollen; Table S2: Instrument/devices, sample treatment, UPLC elution program, and MS/MS conditions; Table S3: Mean and confidence interval (95%) values in μg/kg obtained for the GSLs in commercial samples. The GLSs not detected in those samples (GIB, GER and PRO) were not included; Table S4: Frequency and concentration range of each GSL from MO apiary; Table S5: Mean and confidence interval (95%) values in μg/kg obtained for the GSLs in the samples from MO apiary. The GLSs not detected in those samples (GIB and GTL) were not included; Table S6: Frequency and concentration range of each GSL from PI apiary; Table S7: Mean and confidence interval (95%) values in μg/kg obtained for the GSLs in the samples from PI apiary. The GLSs not detected in those samples (GIB) were not included; Table S8: Frequency and concentration range of each GSL from TN apiary; Table S9: Mean and confidence interval (95%) values in μg/kg obtained for the GSL in the samples from TN apiary. The GSLs not detected in those samples (GIB, PRO, GRA and 4-OH) were not included; Table S10 Frequency and concentration range of each GSL from FH apiary; Table S11: Mean and confidence interval (95%) values in μg/kg obtained for the GSLs in the samples from FH apiary. The GSLs not detected in those samples (GIB, 4-ME and GTL) were not included; Table S12: Weights of the first three canonical variables for the 83 samples; Table S13: Mean values of the canonical variables for the 83 samples; Table S14: Number of observations and percentage classified in each group using canonical discriminant analysis; Table S15: Weights of the first three canonical variables for the 72 samples (origin); Table S16: Mean values of the canonical variables for the 72 samples (origin); Table S17: Number of observations and percentage classified in each apiary using canonical discriminant analysis; Table S18: Weights of the first two canonical variables for the 72 samples (harvesting periods); Table S19: Mean values of the canonical variables for the 72 samples (harvesting periods); Table S20: Number of observations and percentage classified in each group (harvesting period) using canonical discriminant analysis.
