# Supplementary material for: Glucosinolates as Markers of the Origin and Harvesting Period for Discrimination of Bee Pollen by UPLC-MS/MS
Source: Foods. 2022 May 17;11(10):1446. doi: 10.3390/foods11101446 (PMC9141840; doi:10.3390/foods11101446)

Supplementary Material

Glucosinolates as Markers of the Origin and Harvesting Period for Discrimination of Bee Pollen by UPLC-MS/MS

Ana M. Ares [1], Jesús A. Tapia [1,2], Amelia V. González-Porto [3], Mariano Higes [3], Raquel Martín-Hernández [3,4] and José Bernal [1,*]

[1] I. U. CINQUIMA, Analytical Chemistry Group (TESEA), Faculty of Sciences, University of Valladolid, 47011 Valladolid, Spain; ana.maria.ares@uva.es (A.M.A.); jesus.tapia@uva.es (J.A.T.)
[2] Department of Statistics and Operations Research, Faculty of Sciences, University of Valladolid, 47011 Valladolid, Spain
[3] Instituto Regional de Investigación y Desarrollo Agroalimentario y Forestal de Castilla La Mancha (IRIAF), Centro de Investigación Apícola y Agroambiental (CIAPA), Camino de San Martín, s/n, 19180 Marchamalo, Spain; avgonzalezp@jccm.es (A.V.G.-P.); mhiges@jccm.es (M.H.); rmhernandez@jccm.es (R.M.-H.).
[4] Instituto de Recursos Humanos para la Ciencia y la Tecnología (INCRECYT-EFS/EC-FSE), Fundación Parque Científico y Tecnológico de Castilla—La Mancha, 02006 Albacete, Spain
* Correspondence: jose.bernal@uva.es; Tel.: +34-98-318-6347

Table S1. Colony of origin and harvesting period for each bee pollen sample.

**Colony**	**Harvesting period**		
	**April-May**	**June**	**July-August**
**C-FH-1**	FH-1	-	-
**C-FH-2**	FH-2	-	-
**C-FH-3**	-	FH-4	FH-6
**C-FH-4**	FH-3	-	-
**C-FH-5**	-	FH-5	FH-7
**C-MO-1**	MO-1	MO-5	MO-10
**C-MO-2**	MO-2	MO-6	-
**C-MO-3**	-	MO-7	-
**C-MO-4**	MO-3	MO-8	MO-11
**C-MO-5**	MO-4	MO-9	MO-12
**C-PI-1**	PI-1	PI-14	
**C-PI-2**	PI-2	PI-15	PI-32
**C-PI-3**	PI-3	PI-16	PI-33
**C-PI-4**	PI-4	PI-17	PI-34
**C-PI-5**	-	PI-18	PI-35
**C-PI-6**	-	PI-19	PI-36
**C-PI-7**	PI-5	PI-20	-
**C-PI-8**	PI-6	PI-21	PI-37
**C-PI-9**	PI-7	-	-
**C-PI-10**	PI-8	PI-22	PI-38
**C-PI-11**	-	PI-23	PI-39
**C-PI-12**	PI-9	-	-
**C-PI-13**	PI-10	PI-24	PI-40
**C-PI-14**	-	PI-25	-
**C-PI-15**	PI-11	PI-26	PI-41
**C-PI-16**	-	PI-27	PI-42
**C-PI-17**	-	PI-28	PI-43
**C-PI-18**	PI-12	PI-29	PI-44
**C-PI-19**	PI-13	PI-30	PI-45
**C-PI-20**	-	PI-31	-

Table S1. Continued.

Colony	Harvesting period		
	**April-May**	**June**	**July-August**
**C-TN-1**	-	TN-1	TN-6
**C-TN-2**	-	TN-2	-
**C-TN-3**	-	TN-3	TN-7
**C-TN-4**	-	TN-4	-
**C-TN-5**	-	TN-5	TN-8

Table S2. Instrument/devices, Sample treatment, UPLC elution program and MS/MS conditions.

**Instruments/devices**	Syringe filters (17 mm, Nylon 0.45 μm) were purchased from Nalgene (Rochester, NY, USA), and ultrapure water was obtained from Millipore Milli-RO plus and Milli-Q systems (Bedford, MA, USA). An Eppendorf Centrifuge 5810R (Hamburg, Germany), an R-210/215 rotary evaporator 109 (Buchi, Flawil, Switzerland), Bond Elut NH2 (3 mL with 500 mg of sorbent) SPE cartridges from Agilent Technologies (Palo Alto, CA, USA), a 10-port Visiprep vacuum manifold (Supelco, St. Louis, MO, USA), a Moulinette chopper device from Moulinex (Paris, France), and a Vibromatic mechanical shaker and a drying oven both from J.P. Selecta S.A. (Barcelona, Spain) were used for the extractions.
**Sample treatment**	The preparation of the samples was performed according to [23]. Briefly, 1 g of bee pollen sample and 10 mL of heated water (70 °C) were transferred to a centrifuge tube. The mixture was shaken for 10 min at maximum speed (960 oscillations per minute) in a Vibromatic and then centrifuged for 10 min at 4 °C and 10414g. The supernatant was collected and loaded onto a Bond Elut NH_2 SPE cartridge (previously conditioned with 5 mL of methanol and 5 mL of 1% acetic acid in water (v/v)) at about 1 mL/min by means of a suction system. The SPE cartridge was then washed with 5 mL of 5% acetic acid in methanol (v/v); the rinse was discarded, and, after 5 min of drying time, the analytes were eluted with 10 mL of freshly prepared 2% solution of ammonium hydroxide in methanol. The resulting solution was evaporated to dryness at 40 °C in a rotary evaporator; the dry residue was reconstituted with 1 mL of ultrapure water, filtered through a nylon 0.45-μm filter, and injected into the UHPLC-QTOF system.
**UPLC elution program**	It was employed the same program as optimized in [18]. The mobile phase was composed of 0.1% (v/v) formic acid in water (solvent A) and 0.1% (v/v) formic acid in acetonitrile (solvent B) applied at a flow rate of 0.2 mL/ min in the following gradient mode: (**i**) 0.0–2.0 min (A:B, 100:0, v/v); (**ii**) 2.0–2.1 min (A:B, 90:10, v/v); (**iii**) 2.1–6.0 min (A:B, 90:10, v/v); (**iv**) 6.0–6.1 min (A:B, 50:50, v/v); (**v**) 6.1–7.5 min (A:B, 35:65, v/v); (**vi**) 7.5–7.6 min (A:B, 10:90, v/v); (**vii**) 7.6–9.5 min (A:B, 10:90, v/v); (**viii**) 9.5–11.0 min (A:B, 35:65, v/v); (**ix**) 11.0–12.5 min (A:B, 100:0, v/v); (x) 12.5–15.0 min (A:B, 100:0, v/v).
**MS/MS conditions**	Capillary voltage, −2250 V; drying gas flow, 12 L/min; drying gas temperature, 220 °C; nebulizer pressure, 2 bar. GSLs were quantified by generating extracted ion chromatograms with the precursor ions; meanwhile the most relevant fragments for each precursor ion were also used to confirm the presence of each GSL. These MS/MS experiments were carried out by using an isolation width of 10 m/z and a collision energy of 40 eV.

Table S3. Mean and confident interval (95%) values in µg/kg (dry weight) obtained for the GSLs in commercial (CO) samples. The GLSs not detected in those samples (GIB, PRO and GER) were not included.

MUESTRA	SIN	EPI	GRA	GNA	ALY	4-OH	GBN	GTL	GBC	NAS	4-ME	NEO
**CO-1**	<LOD	557±10	249±11	1216±70	<LOD	4193±53	<LOQ	286±3	<LOQ	45±0	<LOD	<LOD
**CO-2**	<LOD	<LOQ	<LOD	231±5	<LOD	639±141	<LOQ	228±17	<LOD	<LOQ	<LOD	<LOD
**CO-3**	<LOD	<LOD	69±5	60±10	<LOD	820±58	<LOD	<LOD	<LOD	<LOD	<LOD	<LOD
**CO-4**	<LOD	57±4	<LOD	84±7	<LOD	1667±81	<LOD	158±5	419±0	623±30	<LOQ	<LOD
**CO-5**	<LOD	<LOD	<LOD	<LOQ	<LOQ	1159±35	<LOQ	<LOD	<LOD	<LOD	<LOD	<LOD
**CO-6**	<LOD	<LOD	189±2	98±9	<LOD	899±63	<LOD	<LOD	<LOD	<LOD	<LOD	<LOD
**CO-7**	<LOD	<LOD	<LOD	<LOQ	<LOD	1199±87	<LOD	23±1	<LOD	<LOD	<LOD	<LOD
**CO-8**	<LOD	<LOD	<LOD	<LOD	<LOD	702±47	<LOD	<LOD	<LOD	<LOD	<LOD	<LOQ
**CO-9**	<LOD	<LOD	<LOD	49±7	<LOD	953±103	<LOD	<LOQ	<LOD	<LOD	<LOQ	<LOD
**CO-10**	<LOD	<LOD	170±2	304±0	<LOD	1216±61	<LOD	24±0	<LOD	<LOQ	<LOQ	<LOD
**CO-11**	92±6	365±1	<LOD	245±5	<LOD	1291±85	<LOD	1593±41	73±3	23±1	<LOD	<LOD

Table S4. Frequency and concentration range of each GSL from MO apiary.

GSL	Frequency[A] (%)	Concentration Range* (μg/kg; Dry Weight)
**GIB**	0	<LOD
**PRO**	42	299-4377
**SIN**	17	1374-1851
**EPI**	25	<LOQ
**GRA**	33	47-1036
**GNA**	58	34-359
**ALY**	33	266-3078
**4-OH**	42	195-3067
**GBN**	83	52-6595
**GTL**	0	<LOD
**GER**	42	36-102
**GBC**	33	98-453
**NAS**	50	50-4724
**4-ME**	8	<LOQ
**NEO**	75	51-4367

[A](number of samples in which a GLSs residue was detected/total number of samples (n = 12)) × 100. *Concentrations over LOQ.

Table S5. Mean and confident interval (95%) values in μg/kg (dry weight) obtained for the GSLs in the samples from MO apiary. The GLSs not detected in those samples (GIB and GTL) were not included.

SAMPLE	PRO	SIN	EPI	GRA	GNA	ALY	4-OH	GBN	GER	GBC	NAS	4-ME	NEO
**MO-1**	1266±4	1851±45	<LOD	270±11	77±6	1261±1	772±9	1968±30	37±1	98±5	859±30	<LOD	150±1
**MO-2**	2832±57	1374±29	<LOD	341±1	359±6	3078±113	285±3	5266±23	102±0	<LOD	4724±51	<LOD	959±18
**MO-3**	4377±64	<LOD	<LOD	1036±8	161±0	937±30	3067±5	2937±167	84±1	167±7	2324±63	<LOD	4367±107
**MO-4**	299±11	<LOD	<LOQ	47±0	34±1	266±5	491±22	580±14	<LOQ	<LOD	132±6	<LOD	<LOD
**MO-5**	<LOQ	<LOD	<LOD	<LOD	50±1	<LOD	<LOD	555±33	<LOD	453±14	997±9	<LOD	752±8
**MO-6**	<LOD	<LOD	<LOD	<LOD	<LOD	<LOD	<LOD	91±2	<LOD	<LOD	<LOD	<LOD	271±8
**MO-7**	<LOD	<LOD	<LOD	<LOD	<LOD	<LOD	<LOD	181±6	<LOD	<LOD	<LOD	<LOD	107±3
**MO-8**	<LOD	<LOD	<LOQ	<LOD	334±10	<LOD	195±0	102±3	<LOD	111±9	50±2	<LOQ	401±4
**MO-9**	<LOD	<LOD	<LOD	<LOD	110±2	<LOD	<LOD	6595±49	<LOD	<LOD	<LOD	<LOD	<LOD
**MO-10**	<LOD	<LOD	<LOD	<LOD	<LOD	<LOD	<LOD	<LOD	36±1	<LOD	<LOD	<LOD	51±0
**MO-11**	<LOD	<LOD	<LOQ	<LOD	<LOD	<LOD	<LOD	<LOD	<LOD	<LOD	<LOD	<LOD	153±5
**MO-12**	<LOD	<LOD	<LOD	<LOD	<LOD	<LOD	<LOD	52±3	<LOD	<LOD	<LOD	<LOD	<LOD

Table S6. Frequency and concentration range of each GSL from PI apiary.

GSL	Frequency[A] (%)	Concentration Range * (μg/kg; Dry Weight)
**GIB**	0	<LOD
**PRO**	36	137-6690
**SIN**	44	84-2721
**EPI**	11	41-55
**GRA**	29	58-1172
**GNA**	62	27-1354
**ALY**	29	143-7916
**4-OH**	36	105-1539
**GBN**	76	31-8470
**GTL**	13	22-76
**GER**	36	34-126
**GBC**	27	39-1467
**NAS**	44	21-9936
**4-ME**	33	109-1664
**NEO**	60	29-3928

[A](number of samples in which a GLSs residue was detected/total number of samples (n = 45)) × 100; * Concentrations over LOQ.

Table S7. Mean and confident interval (95%) values in µg/kg (dry weight) obtained for the GSLs in the samples from PI apiary. The GLSs not detected in those samples (GIB) was not included.

SAMPLE	PRO	SIN	EPI	GRA	GNA	ALY	4-OH	GBN	GTL	GER	GBC	NAS	4-ME	NEO
**PI-1**	469±6	392±4	<LOQ	60±0	72±1	692±2	105±0	1939±31	<LOD	<LOQ	<LOD	552±25	<LOD	<LOD
**PI-2**	162±6	626±21	<LOD	62±5	27±1	232±0	<LOD	344±1	<LOD	<LOD	39±1	239±11	<LOD	34±3
**PI-3**	615±14	602±7	<LOD	79±1	63±1	711±13	158±3	1782±5	<LOD	<LOQ	42±0	555±6	<LOD	54±2
**PI-4**	1827±44	2665±188	<LOD	463±24	86±5	865±28	<LOD	1962±172	<LOQ	<LOQ	74±20	1782±176	118±5	<LOD
**PI-5**	4335±118	2158±15	<LOD	598±1	816±16	7916±105	<LOD	6158±65	<LOD	116±2	<LOD	9505±25	<LOD	203±1
**PI-6**	519±14	471±7	<LOD	68±3	70±3	740±10	<LOD	1565±33	<LOD	<LOQ	<LOD	315±9	<LOD	<LOD
**PI-7**	834±12	642±25	<LOD	101±1	77±1	708±14	139±4	2064±42	<LOD	<LOQ	56±2	517±384	<LOD	49±1
**PI-8**	679±14	327±4	<LOD	122±1	128±3	497±369	154±2	1729±9	<LOD	65±5	<LOQ	490±7	<LOD	66±1
**PI-9**	336±7	748±19	<LOD	58±4	30±1	200±3	<LOD	234±171	<LOD	<LOD	<LOQ	265±2	<LOD	1342±19
**PI-10**	722±20	157±5	<LOD	473±3	254±4	2017±24	<LOQ	1805±13	<LOD	35±0	<LOD	430±8	<LOD	946±702
**PI-11**	6690±137	2721±57	<LOD	1172±8	322±10	3866±43	<LOD	8470±47	<LOD	126±18	102±1	9936±0	277±1	3928±36
**PI-12**	414±8	475±14	<LOQ	61±2	43±0	374±9	<LOD	820±36	<LOD	<LOQ	<LOD	195±5	<LOD	<LOD
**PI-13**	294±1	<LOQ	41±1	62±0	27±0	143±2	<LOD	257±7	<LOD	<LOQ	<LOD	61±0	<LOD	<LOD
**PI-14**	<LOD	<LOD	<LOD	<LOD	75±1	<LOD	<LOD	34±1	<LOD	<LOD	<LOQ	<LOD	130±1	<LOD
**PI-15**	<LOQ	<LOQ	<LOD	<LOD	<LOQ	<LOD	<LOD	110±1	<LOD	<LOD	<LOD	<LOD	<LOQ	158±0
**PI-16**	<LOD	84±4	<LOD	<LOD	130±2	<LOD	201±4	<LOD	<LOQ	<LOD	<LOD	90±0	117±2	<LOD
**PI-17**	<LOD	516±1	<LOD	<LOD	69±2	<LOD	171±22	<LOD	<LOD	<LOD	<LOD	<LOD	<LOQ	53±3

**PI-18**	<LOD	<LOD	<LOQ	<LOD	469±7	<LOD	218±15	76±6	<LOD	<LOD	<LOD	<LOD	<LOQ	95±34
**PI-19**	<LOD	<LOD	<LOD	<LOD	92±2	<LOD	235±8	643±11	<LOD	<LOD	<LOQ	61±2	203±1	<LOD
**PI-20**	<LOD	<LOD	<LOD	<LOD	<LOD	<LOD	<LOD	<LOQ	<LOD	<LOD	<LOD	<LOD	<LOD	137±0
**PI-21**	<LOD	<LOD	<LOD	<LOD	<LOD	<LOD	<LOD	<LOD	<LOD	<LOD	<LOD	<LOD	<LOD	<LOD
**PI-22**	<LOD	<LOD	<LOD	<LOD	<LOD	<LOD	<LOD	699±17	<LOQ	47±1	<LOD	<LOD	<LOD	<LOD
**PI-23**	<LOD	<LOD	<LOD	<LOD	295±5	<LOD	<LOD	60±0	<LOD	<LOD	<LOD	<LOQ	<LOD	<LOD
**PI-24**	<LOD	<LOD	<LOD	<LOD	<LOD	<LOD	<LOD	54±0	<LOD	<LOQ	<LOD	<LOD	<LOD	<LOQ
**PI-25**	<LOD	<LOD	<LOD	<LOD	<LOQ	<LOD	<LOD	<LOQ	<LOD	<LOD	<LOD	<LOD	<LOQ	<LOQ
**PI-26**	<LOQ	<LOD	<LOD	<LOD	62±3	<LOD	415±1	31±1	<LOD	36±1	<LOD	49±3	165±4	133±1
**PI-27**	137±6	<LOD	<LOD	<LOD	558±5	<LOD	1121±21	2732±1	76±4	<LOD	<LOD	<LOD	663±20	<LOD
**PI-28**	<LOD	706±22	55±3	<LOD	1354±50	<LOD	<LOD	41±1	<LOQ	<LOD	207±7	<LOD	1664±7	<LOD
**PI-29**	<LOD	292±6	<LOD	<LOD	90±0	<LOD	<LOD	336±19	<LOD	<LOD	<LOD	<LOD	<LOD	77±3
**PI-30**	<LOD	590±6	<LOD	<LOD	400±12	<LOD	296±2	60±3	<LOD	<LOD	1467±14	448±8	109±7	187±6
**PI-31**	<LOD	<LOD	<LOD	<LOD	189±7	<LOD	<LOD	364±15	<LOD	<LOD	<LOD	<LOD	<LOD	54±5
**PI-32**	<LOD	<LOD	<LOD	<LOD	<LOD	<LOD	<LOD	106±4	<LOD	<LOD	<LOD	<LOD	<LOQ	<LOD
**PI-33**	<LOD	<LOD	<LOD	<LOD	<LOD	<LOD	<LOD	466±28	22±4	<LOD	<LOD	<LOD	109±1	<LOD
**PI-34**	<LOD	<LOD	<LOD	<LOD	<LOD	<LOD	<LOD	<LOD	<LOD	<LOD	<LOD	<LOD	<LOD	622±8
**PI-35**	<LOD	<LOD	<LOD	<LOD	<LOD	<LOD	<LOD	<LOD	<LOD	<LOD	<LOD	<LOD	<LOD	974±34
**PI-36**	<LOD	121±3	<LOD	<LOD	160±4	<LOD	<LOD	<LOD	<LOD	<LOD	<LOD	45±0	<LOD	685±3

**PI-37**	<LOD	<LOD	<LOD	<LOD	<LOD	<LOD	<LOD	<LOQ	<LOD	<LOQ	<LOD	21±0	<LOD	<LOD
**PI-38**	<LOD	<LOD	<LOD	<LOD	<LOD	<LOD	<LOD	<LOD	<LOD	<LOD	<LOD	<LOD	<LOD	29±0
**PI-39**	<LOD	<LOD	<LOD	<LOD	<LOD	<LOD	<LOD	<LOD	<LOD	34±6	<LOD	<LOD	<LOD	<LOD
**PI-40**	<LOD	<LOD	<LOD	<LOD	<LOD	<LOD	<LOD	<LOQ	<LOD	<LOD	<LOD	<LOD	<LOD	29±1
**PI-41**	<LOD	<LOD	<LOD	<LOD	<LOD	<LOD	143±2	<LOD	<LOD	<LOD	<LOD	<LOD	<LOD	1272±44
**PI-42**	<LOD	<LOD	<LOD	<LOD	<LOD	<LOD	891±5	<LOQ	<LOD	<LOD	63±1	<LOD	<LOD	<LOD
**PI-43**	<LOD	<LOD	<LOD	<LOD	<LOD	<LOD	1539±24	117±6	<LOD	<LOD	<LOD	<LOD	<LOD	120±3
**PI-44**	<LOD	<LOD	<LOD	<LOD	<LOD	<LOD	1182±22	<LOD	<LOD	<LOD	<LOD	<LOD	<LOD	567±2
**PI-45**	<LOD	<LOD	<LOD	<LOD	<LOD	<LOD	<LOD	<LOQ	<LOD	<LOD	<LOD	<LOD	<LOD	70±1

Table S8. Frequency and concentration range of each GSL from TN apiary.

GSL	Frequency[A] (%)	Concentration Range* (μg/kg; Dry Weight)
**GIB**	0	<LOD
**PRO**	0	<LOD
**SIN**	13	<LOQ
**EPI**	25	<LOQ
**GRA**	0	<LOD
**GNA**	25	162-411
**ALY**	13	<LOQ
**4-OH**	0	<LOD
**GBN**	38	79-221
**GTL**	13	<LOQ
**GER**	13	152
**GBC**	25	155-547
**NAS**	25	76-91
**4-ME**	38	346
**NEO**	38	36-48

[A](number of samples in which a GLSs residue was detected/total number of samples (*n* = 8)) × 100. *Concentrations over LOQ.

Table S9. Mean and confident interval (95%) values in µg/kg (dry weight) obtained for the GSL in the samples from TN apiary. The GSLs not detected in those samples (GIB, PRO, GRA and 4-OH) were not included.

MUESTRA	SIN	EPI	GNA	ALY	GBN	GTL	GER	GBC	NAS	4-ME	NEO
**TN-1**	<LOD	<LOQ	411±1	<LOD	79±9	<LOD	<LOD	155±12	76±4	<LOQ	48±2
**TN-2**	<LOD	<LOD	162±21	<LOD	221±52	<LOQ	<LOD	547±197	91±7	<LOQ	<LOQ
**TN-3**	<LOD	<LOD	<LOD	<LOQ	79±3	<LOD	<LOD	<LOD	<LOD	<LOD	36±3
**TN-4**	<LOD	<LOD	<LOD	<LOD	<LOQ	<LOD	<LOD	<LOD	<LOD	<LOD	<LOD
**TN-5**	<LOD	<LOQ	<LOD	<LOD	<LOD	<LOD	152±0	<LOD	<LOD	<LOD	<LOD
**TN-6**	<LOQ	<LOQ	<LOD	<LOD	<LOQ	<LOD	<LOD	<LOD	<LOD	<LOD	<LOD
**TN-7**	<LOD	<LOD	<LOD	<LOD	<LOQ	<LOD	<LOD	<LOD	<LOD	<LOD	<LOD
**TN-8**	<LOD	<LOD	<LOD	<LOD	<LOD	<LOD	<LOD	<LOD	<LOD	346±0	<LOD

Table S10. Frequency and concentration range of each GSL from FH apiary.

GSL	Frequency[A] (%)	Concentration Range* (μg/kg; Dry Weight)
**GIB**	0	<LOD
**PRO**	43	130-1361
**SIN**	14	478
**EPI**	43	<LOQ
**GRA**	29	184
**GNA**	43	30-131
**ALY**	43	157-1018
**4-OH**	29	166-3370
**GBN**	43	548-1200
**GTL**	0	<LOD
**GER**	14	<LOQ
**GBC**	14	194
**NAS**	86	99-1497
**4-ME**	0	<LOD
**NEO**	71	35-5562

[A](number of samples in which a GLSs residue was detected/total number of samples (n = 7)) × 100. *Concentrations over LOQ.

Table S11. Mean and confident interval (95%) values in µg/kg (dry weight) obtained for the GSLs in the samples from FH apiary. The GSLs not detected in those samples (GIB, 4-ME and GTL) were not included.

SAMPLE	PRO	SIN	EPI	GRA	GNA	ALY	4-OH	GBN	GER	GBC	NAS	NEO
**FU-1**	1361±47	478±14	<LOD	184±0	131±6	1018±29	3370±86	904±2	<LOD	<LOD	279±4	5562±84
**FU-2**	130±0	<LOD	<LOQ	<LOD	<LOQ	157±2	<LOD	548±11	<LOD	<LOD	99±2	<LOQ
**FU-3**	365±5	<LOD	<LOQ	<LOQ	30±1	283±2	166±18	1200±24	<LOQ	<LOD	218±2	<LOD
**FU-4**	<LOD	<LOD	<LOD	<LOD	<LOD	<LOD	<LOD	<LOD	<LOD	<LOD	<LOQ	132±2
**FU-5**	<LOD	<LOD	<LOQ	<LOD	<LOD	<LOD	<LOD	<LOD	<LOD	194±1	1497±88	426±8
**FU-6**	<LOD	<LOD	<LOD	<LOD	<LOD	<LOD	<LOD	<LOD	<LOD	<LOD	<LOD	35±1
**FU-7**	<LOD	<LOD	<LOD	<LOD	<LOD	<LOD	<LOD	<LOD	<LOD	<LOD	<LOQ	<LOD

Table S12. Weights of the first three canonical variables for the 83 samples.

Variable	Can1	Can2	Can3
**PRO_1**	-0.029047313	0.016096285	0.049168694
**PRO_2**	0.002967934	0.153952971	0.008411896
**PRO_3**	0.021118629	-0.171696190	-0.061440690
**SIN_1**	-0.053151810	0.022857796	0.029459475
**SIN_2**	0.015057546	-0.010756261	0.131756633
**SIN_3**	0.040197830	-0.005046324	-0.162544499
**EPI_1**	-0.582930260	0.675436703	-1.306376552
**EPI_2**	0.166944122	0.733024926	-0.927492934
**EPI_3**	0.403538184	-1.405024433	2.229260254
**GRA_1**	-0.225422837	-0.337479147	-0.120504978
**GRA_2**	-0.468469099	-0.467611206	-0.144537582
**GRA_3**	0.707437189	0.804334119	0.261912824
**GNA_1**	-0.029831969	0.373605104	-0.062751251
**GNA_2**	-0.092247455	0.436590348	-0.061735658
**GNA_3**	0.124155346	-0.809705096	0.125962444
**ALY_1**	0.094260396	0.023241338	-0.136860563
**ALY_2**	0.092984425	0.080691769	-0.275359760
**ALY_3**	-0.187400719	-0.103817812	0.413809837
**4_OH_1**	-0.014177858	0.002349057	0.006244149
**4_OH_2**	-0.036351288	0.000981165	0.008906508
**4_OH_3**	0.053275145	-0.002501512	-0.014815507
**GBN_1**	0.022578965	-0.072821570	0.096316975
**GBN_2**	0.004767189	-0.011002234	0.098473225
**GBN_3**	-0.027374258	0.082880002	-0.194274574
**GTL_1**	-0.372029112	-0.298840960	0.153577452

**GTL_2**	-0.102526182	-0.991561539	0.303585402
**GTL_3**	0.490818188	1.279180896	-0.455472084
**GER_1**	-0.197759955	0.296168033	0.573327514
**GER_2**	-0.097211863	0.780934963	0.451198252
**GER_3**	0.292157883	-1.083347830	-1.012017517
**GBC_1**	0.038230513	-0.066631118	0.029027182
**GBC_2**	0.146430492	-0.273044898	0.114322420
**GBC_3**	-0.185790368	0.336828620	-0.141800512
**NAS_1**	-0.013098735	-0.011966354	-0.025735045
**NAS_2**	0.013444044	0.014822097	0.029096033
**NAS_3**	0.001481710	-0.002270233	-0.004064490
**4_ME_1**	0.226573498	-0.496440964	-0.076891914
**4_ME_2**	0.025263631	-0.109729842	-0.224630635
**4_ME_3**	-0.255027123	0.608698160	0.304750980
**NEO_1**	-0.013765178	-0.085644742	0.053401247
**NEO_2**	-0.031917217	-0.122330458	0.068038468
**NEO_3**	0.043939100	0.207736089	-0.120487023

Table S13. Mean values of the canonical variables 1 and 2 for the 83 samples.

Group	Nº Observations	Variable	Mean
FH	7	**Can1**	-1.0427355
		**Can2**	-1.5899360
		**Can 3**	-2.3746905
MO	12	**Can1**	-1.2987433
		**Can2**	-2.3660896
		**Can 3**	0.8481109
PI	45	**Can1**	-0.6077352
		**Can2**	1.0672124
		**Can 3**	0.0129179
TN	8	**Can1**	-0.9423825
		**Can2**	-0.5117047
		**Can 3**	0.6974254
CO	11	**Can1**	5.2519283
		**Can2**	-0.4007541
		**Can 3**	0.0258906

Table S14. Number of observations and percentage classified in each group using canonical discriminant analysis.

Origin	FH	MO	PI	TN	CO	Total
**FH**	4	1	0	2	0	7
%	57.14	14.29	0.00	28.57	0.00	100.00
**MO**	0	11	0	1	0	12
%	0.00	91.67	0.00	8.33	0.00	100.00
**PI**	0	0	32	10	3	45
%	0.00	0,000	71.11	22.22	6.67	100.00
**TN**	0	0	0	8	0	8
%	0.00	0.00	0.00	100.00	0.00	100.00
**CO**	0	0	0	0	11	11
%	0.00	0.00	0.00	0.00	100.00	100.00
**Total**	4	12	32	21	14	83
	4.82	14.46	38.55	25.30	16.87	100.00

Table S15. Weights of the first three canonical variables for the 72 samples (origin).

Variable	Can1	Can2	Can3
**PRO_1**	-0.078745945	-0.116207143	0.121683634
**PRO_2**	0.127590164	-0.154520816	0.076472635
**PRO_3**	-0.049191393	0.275374820	-0.197026038
**SIN_1**	-0.111818356	-0.008649097	0.072056303
**SIN_2**	-0.108485741	-0.201103259	0.026351561
**SIN_3**	0.226762108	0.209684686	-0.101693079
**EPI_1**	1.789579869	-0.813021601	-1.912587191
**EPI_2**	3.270330127	0.733363327	0.019271430
**EPI_3**	-5.082371063	0.068897542	1.823497872
**GRA_1**	1.208119911	0.555488170	-0.094246958
**GRA_2**	0.169633144	1.133910857	0.510230481
**GRA_3**	-1.403109611	-1.699194905	-0.424533089
**GNA_1**	0.675320477	0.742149277	0.020598370
**GNA_2**	0.615895505	0.322197264	-0.128720350
**GNA_3**	-1.285343203	-1.063587445	0.113914054
**ALY_1**	0.029711270	0.028684922	-0.180132726
**ALY_2**	0.031536749	0.089802646	-0.267282265
**ALY_3**	-0.057128787	-0.119667751	0.447992926
**4_OH_1**	0.007623856	0.047557136	0.059431932
**4_OH_2**	-0.040703973	-0.021372745	0.029864601
**4_OH_3**	0.034010811	-0.026943455	-0.089527495
**GBN_1**	-0.171788584	-0.086374718	0.013865749
**GBN_2**	-0.074148065	-0.057677099	0.014472501
**GBN_3**	0.244638563	0.144318114	-0.027903843
**GTL_1**	3.435596513	4.811908653	-0.166599369
**GTL_2**	0.082058717	1.344653238	-0.168433114
**GTL_3**	-3.507478458	-6.085263345	0.298816621

**GER_1**	0.481744379	-1.197332050	0.902250394
**GER_2**	0.925328929	-1.006774676	0.675306355
**GER_3**	-1.417511691	2.180542205	-1.566404109
**GBC_1**	-0.102153412	0.003737694	0.022095882
**GBC_2**	-0.512276796	-0.295470632	0.030253232
**GBC_3**	0.609992802	0.292287398	-0.050589254
**NAS_1**	-0.004408643	0.008856185	-0.006644458
**NAS_2**	0.005692356	-0.012198799	0.012125342
**NAS_3**	-0.000927427	0.004224894	-0.007445508
**4_ME_1**	-0.453362143	0.248120204	-0.168325744
**4_ME_2**	0.444436857	0.999971883	-0.102166986
**4_ME_3**	0.008175248	-1.259628513	0.270328426
**NEO_1**	-0.129622269	-0.068110374	0.109881240
**NEO_2**	-0.135379020	-0.040639459	0.108501425
**NEO_3**	0.265728523	0.109593534	-0.217093249

Table S16. Mean values of the canonical variables 1 and 3 for the 72 samples (apiary of origin).

Group	Nº Observations	Variable	Mean
FH	7	Can1	-1.5801771
		Can2	0.9612291
		Can 3	-2.2140864
MO	12	Can1	-2.5838455
		Can2	0.7672015
		Can 3	1.0680189
PI	45	Can1	1.2116470
		Can2	0.0771155
		Can 3	0.1027449
TN	8	Can1	-1.5570911
		Can2	-2.4256523
		Can 3	-0.2426428

Table S17. Number of observations and percentage classified in each apiary using canonical discriminant analysis.

Origin	FH	MO	PI	TN	Total
**FH**	5	0	0	2	7
**%**	71.43	0.00	0.00	28.57	100.00
**MO**	0	10	0	2	12
**%**	0.00	83.33	0.00	16.67	100.00
**PI**	3	0	39	3	45
**%**	6.67	0,000	86.67	6.67	100.00
**TN**	0	0	0	8	8
**%**	0.00	0.00	0.00	100.00	100.00
**Total**	8	10	39	15	72
	11.11	13.89	54.17	20.83	100.00

Table S18. Weights of the first two canonical variables for the 72 samples (harvesting periods).

Variable	Can1	Can2
**PRO_1**	-0.080201118	0.042524563
**PRO_2**	0.055348265	0.146737661
**PRO_3**	0.022739485	-0.176307024
**SIN_1**	-0.050814301	0.110659318
**SIN_2**	-0.216326233	0.056173528
**SIN_3**	0.267766485	-0.165866447
**EPI_1**	4.137764804	1.231739870
**EPI_2**	-0.595786849	-0.501886895
**EPI_3**	-3.267159906	-0.704273036
**GRA_1**	-1.228041451	-0.284852906
**GRA_2**	0.696769188	0.233886798
**GRA_3**	0.538952012	-0.007411957
**GNA_1**	0.525335229	-0.059027491
**GNA_2**	0.841944528	0.250136764
**GNA_3**	-1.385212738	-0.185903536
**ALY_1**	0.063254618	-0.037751351
**ALY_2**	-0.033033065	0.047784825
**ALY_3**	-0.020271029	-0.015473271
**_4_OH_1**	0.105084115	-0.011156037
**_4_OH_2**	0.020359864	0.000641472
**_4_OH_3**	-0.127581926	0.008882004
**GBN_1**	-0.062075012	-0.040828744
**GBN_2**	0.047185500	0.038062074
**GBN_3**	0.014457572	0.002441351
**GTL_1**	-0.176279802	2.354322840
**GTL_2**	0.171249741	1.358018936
**GTL_3**	0.199997669	-3.508700398

**GER_1**	-2.456659926	-2.933516584
**GER_2**	-1.796419403	-2.797354996
**GER_3**	4.213306275	5.745429203
**GBC_1**	-0.140745700	-0.100315260
**GBC_2**	-0.587321053	-0.350532939
**GBC_3**	0.732428202	0.452024171
**NAS_1**	-0.016210049	0.001481072
**NAS_2**	0.025092686	-0.003238585
**NAS_3**	-0.010932472	0.002922961
**_4_ME_1**	-0.225535705	0.126673852
**_4_ME_2**	-0.192901959	-0.301996518
**_4_ME_3**	0.416311984	0.173859513
**NEO_1**	-0.022736015	-0.004774465
**NEO_2**	0.026057150	0.029671365
**NEO_3**	-0.001712700	-0.025406925

Table S19. Mean values of the canonical variables 1 and 2 for the 72 samples (harvesting periods).

Origin	Nº Observations	Variable	Mean
**April-May**	**20**	**Can1**	6.2380705
		**Can2**	0.0144656
**June**	**22**	**Can1**	-2.4444886
		**Can2**	-1.3811192
**July-August**	**30**	**Can1**	-2.5319706
		**Can2**	0.9901445

Table S20. Number of observations and percentage classified in each group (harvesting period) using canonical discriminant analysis.

Origin	April-May	June	July-August	Total
**April-May**	20	0	0	20
%	100.00	0.00	0.00	100.00
**June**	0	21	1	22
%	0.00	95.45	4.55	100.00
**July-August**	0	10	20	30
%	0.00	34.48	66,67	100.00
**Total**	20	31	21	72
	27.78	43.06	29,17	100.00

Figure S1. UPLC-MS/MS extracted ion chromatogram obtained in positive mode using the quantification ions (see Table 1) from PI-11 bee pollen sample in which eleven GSLs were detected: PRO (1); SIN (2); GRA (3); GNA (4); ALY (5); GBN (6); GER (17); GBC (8); NAS (9); 4-Me (10); NEO (11). The UPLC-MS/MS conditions are summarized in Subsection 2.4 and Table 1.

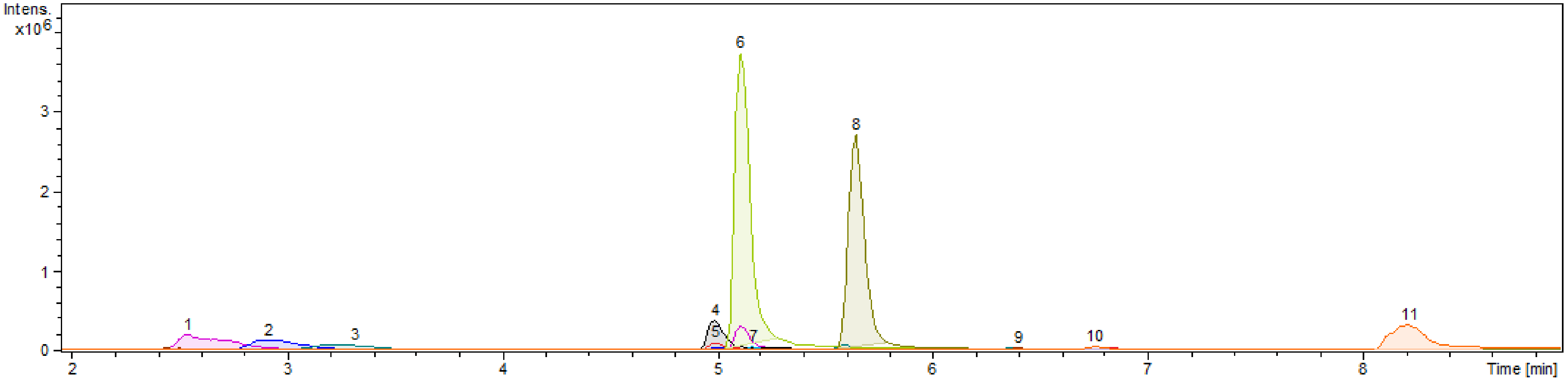

Figure S2. Representation of individual bee pollen samples (see Table 2) from the apiaries (FH, 1; MO, 2; PI, 3; TN, 4) and local markets (5) as function of the first two canonical variables**.**

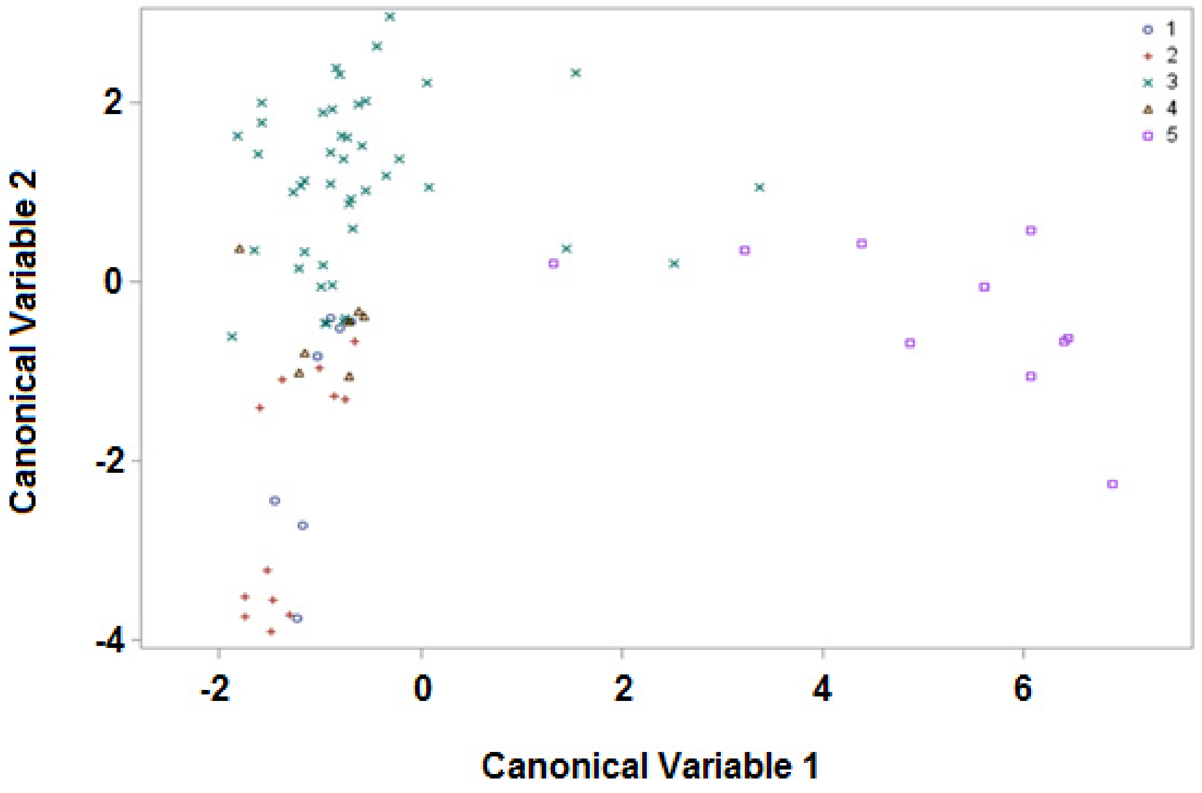

Figure S3. Representation of individual bee pollen samples (see Table 2) from the apiaries (FH, 1; MO, 2; PI, 3; TN, 4) as function of canonical variables 1 and 3**.**

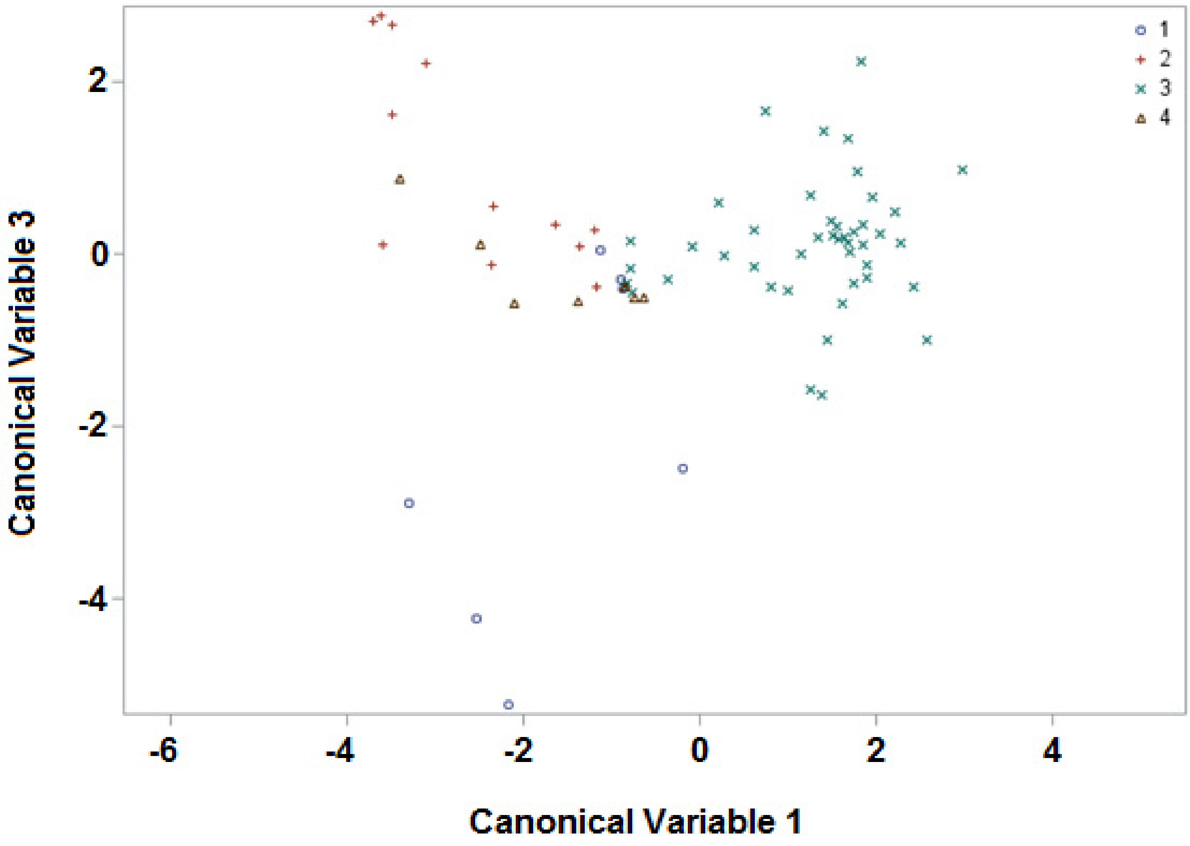

Figure S4. Representation of individual bee pollen samples (see Table 2) from the different harvesting periods (April-May, 1; June, 2; July-August, 3) as function of the first two canonical variables.

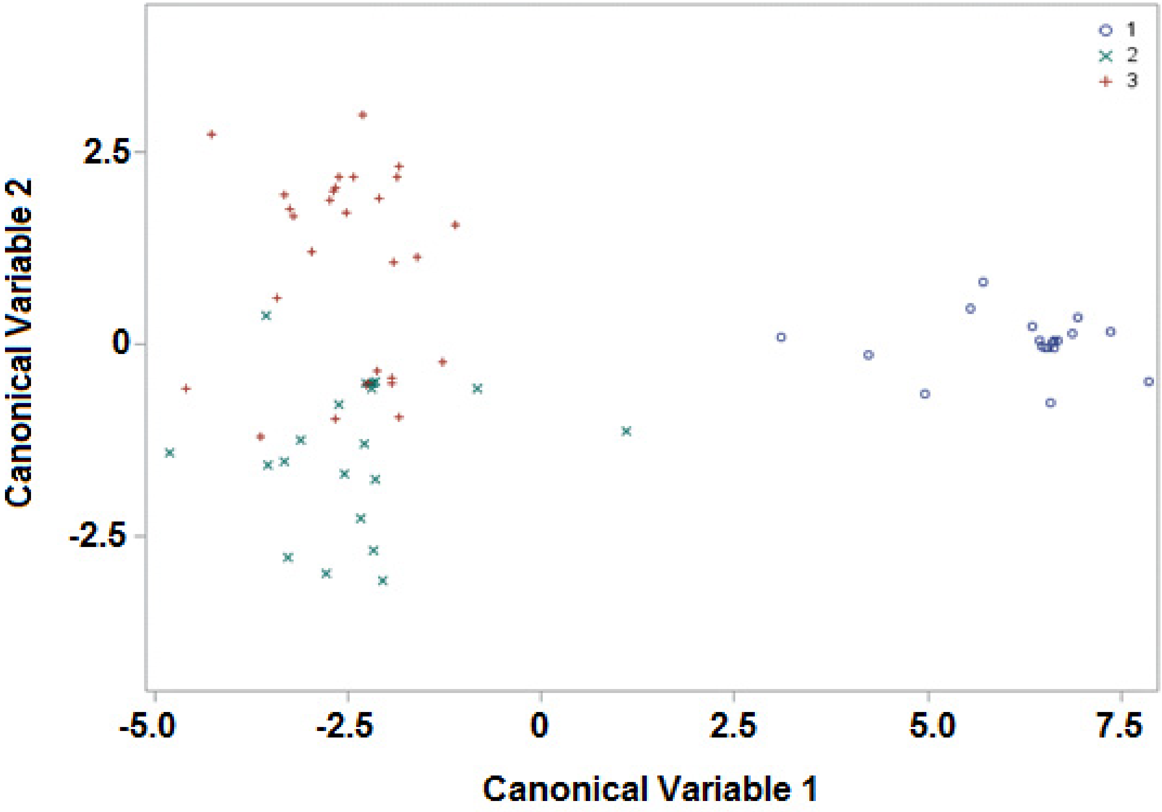

Supplement: Supplementary file 1 [file foods-11-01446-s001.zip › foods-1702179-supplementary.pdf]
